# Mapping the spatial immunometabolic responses of microglia in response to Alzheimer’s pathology

**DOI:** 10.1002/alz.095162

**Published:** 2025-01-09

**Authors:** Kai Saito, Danielle S. Goulding, Sophia H Dimas, Ela Patel, Andy Snipes, Peter T Nelson, Shannon L Macauley, Josh M. Morganti

**Affiliations:** ^1^ University of Kentucky College of Medicine, Sanders‐Brown Center on Aging, Lexington, KY USA

## Abstract

**Background:**

Single cell RNA sequencing has defined multiple transcription states of microglia in the context of AD neuropathology. Growing appreciating for several of these disease‐associated phenotypes are linked with acquisition of altered metabolism, conceptually known as immunometabolism. Despite increasing knowledge in microglial heterogeneity, relatively little is known regarding the spatial distribution of these phenotypes in the context of pathology proximity.

**Method:**

Using 10X Genomics Xenium system, we developed a fully custom 400+ gene panel consisting of probes for deep phenotyping of microglial heterogeneity, in tandem with canonical and rate‐limiting genes associated with multiple cellular metabolic pathways, as well as genes that canonically define neuronal subsets, astrocytes, endothelia, oligodendrocytes, pericytes, and infiltrating immune classes, etc. This panel was employed across three AD‐recapitulating mouse models (n = 2/3 per genotype): PS19 (12 months old), APP/PS1 (12 months old), and 5xFAD (22 months old), and their age‐matched ‘WT’ littermate controls. In parallel, we performed the identical experiments using custom Xenium probe set in age and sex matched human autopsy tissue from the dorsolateral PFC gray matter from cases (n = 2/bin) with low (Braak I/II), middle (Braak III/IV) and high (Braak V/VI) pathological burden.

**Result:**

We demonstrate unique intrinsic relationships of microglial heterogeneity as a function of AD‐associated pathological burden in our mouse models. ‘DAM’‐like microglia displace anatomically‐restricted homeostatic‐associated heterogeneity in as a function of pathology accumulation. In the PS19 model, we demonstrate significant susceptibility of the hippocampal formation to acquire DAM‐like and Interferon‐assocatied microglial phenotypes, whereas in both the APP/PS1 and 5xFAD broader anatomical accumulation was apparent in cortical, hippocampal, and thalamic regions. Using machine learning, we demonstrate that proximity to ThioS+ pathology drives distinctive metabolic shifts in microglial populations, with preferential signatures enriching upon both glycolytic as well as cholesterol metabolism alterations. Further, several of the indices observed in our mouse models were conserved in the human tissue.

**Conclusion:**

Microglial heterogeneity is governed by anatomical locale as well as a function of proximity to AD‐related pathology, highlighting areas of susceptibility, which is correlated with shifts in transcriptional profiles linked with both glycolytic and cholesterol metabolism.

